# Lassa virus RNA detection from suspected cases in Nigeria, 2011-2017

**DOI:** 10.11604/pamj.2019.34.76.16425

**Published:** 2019-10-06

**Authors:** Salu Olumuyiwa Babalola, James Ayorinde Babatunde, Orenolu Mercy Remilekun, Anyanwu Roosevelt Amaobichukwu, Abdullah Mariam Abiodun, Idris Jide, Abdus-Salam Ismail Adeshina, Ihekweazu Chikwe, Omilabu Sunday Aremu

**Affiliations:** 1 Department of Medical Microbiology and Parasitology, College of Medicine, University of Lagos (CMUL), PM.B. 12003, Lagos, Nigeria; 2 Centre for Human and Zoonotic Virology (CHAZVY), Central Research Laboratory, College of Medicine, University of Lagos (CMUL)/Lagos University Teaching Hospital (LUTH), Lagos, Nigeria; 3 Department of Biochemistry, College of Medicine, University of Lagos, PM.B. 12003, Lagos, Nigeria; 4 Honourable Commissioner for Health, Lagos State Ministry of Health, Alausa, Ikeja, Lagos, Nigeria; 5 Epidemiology Unit, Directorate of Disease Control, Lagos State Ministry of Health, Alausa, Ikeja, Lagos, Nigeria; 6 Nigerian Centre for Disease Control (NCDC), Plot 801, Ebitu Ukiwe Street, Jabi, Abuja, Nigeria

**Keywords:** Lassa virus (LASV), public health burden, viral haemorrhagic fever (VHF), endemicity, reverse transcription-polymerase chain reaction (RT-PCR), sensitization and health education

## Abstract

**Introduction:**

The diagnosis of Lassa fever is crucial to confirm cases, as well as to control/prevent nosocomial and community-based transmission and initiation of treatment, which is still limited in the country. Thus, we aimed at providing some information on the laboratory detection of Lassa from suspected cases in Nigeria.

**Methods:**

This was a retrospective study of seasonal Lassa fever outbreaks data from 1,263 samples analyzed using Reverse Transcription-Polymerase Chain Reaction (RT-PCR) at the Virology Research Laboratory, College of Medicine, University of Lagos/Lagos University Teaching Hospital between year 2011 and 2017. Data were analyzed using the 21^st^ edition of SPSS statistical software (2015).

**Results:**

The RT-PCR test confirmed the presence of Lassa in 112 (8.9%) comprising 61 (54.4%) males, 48 (42.9%) females and 3 (2.7%) individuals without gender information. Those aged between 18 and 49 years were mostly affected. There was a decline in the detection of Lassa from 4.7% in 2011/2012 to less than 1% by the 2014/2015. However, during the 2015/2016 and 2016/2017 seasons the detection rates increased to 10.4% and 15.1% respectively. The Northern region of Nigeria reported high confirmed cases of Lassa. The South Western region also witnessed an increased Lassa fever positivity rate of 13.4% of which Lagos and Ogun states being the focal state of Lassa activity in the region.

**Conclusion:**

These established the need for heightening the continued surveillance for Lassa as well as the establishment of other testing facilities within these endemic regions for prompt diagnosis of Lassa fever.

## Introduction

Lassa virus (LASV), a member of the family; Arenaviridae is the causative agent of Lassa fever (LF), or Lassa hemorrhagic fever which is characterized by a common acute and often fatal febrile syndrome endemic to West Africa [1-4]. Lassa fever, an infectious illness is estimated to affect 150,000 to 300,000 people in West Africa causing up to 5,000 deaths per year and is associated with significant morbidity and mortality [3-5]. However, sero-epidemiologic survey had suggested that the number of cases might be much higher, reaching 3 million cases and 67,000 fatalities per year [6]. Overall, the population at risk might include as many as 200 million persons living in a large strip of West Africa from Senegal to Nigeria and beyond [7]. Lassa fever differs from most viral haemorrhagic fevers (VHFs) in that it is endemic to a large geographic area of sub-Saharan Africa. Human cases of LF have been reported in (or imported from) Guinea, Sierra Leone, Liberia, Mali, Burkina Faso, and Nigeria; however, LF outbreaks seem to be restricted to Guinea, Sierra Leone, Liberia (the Mano River Union region), and Nigeria [2,7-9]. The Multimammate rat *Mastomys natalensis*, discovered in 1974 as a natural host and reservoir of lassa virus (LASV) is a commensal rodent ubiquitous in Africa [2,3,10-12]. However, other reservoirs such as *Hylomyscus pamfi* and *Mastomys erythroleucus* had also been discovered to harbor Lassa [3]. Although the routes of LASV infection are poorly characterized and the actual burden of the disease still not fully determined, humans probably get infected by eating contaminated food [13], by inhaling virus-contaminated aerosols [14], or while butchering infected rat meat [15]. Person-to-person transmission of LASV is well documented, mostly in the form of nosocomial outbreaks [13]. Presently, there is no licensed vaccine or immunotherapy available for prevention or treatment of this disease. International transportation to and from Africa had increased dramatically in the last decade, further increasing the risk for infectious disease exportation from endemic areas [16,17]. Unlike the rare outbreaks of Ebola Virus Disease (EVD), LF cases in sub-Saharan Africa are common and occur annually, therefore posing a continuous theoretical threat to other parts of Africa and the world at large.

In West Africa, since Lassa fever presents with signs and symptoms related to numerous other febrile endemic diseases such as malaria, dengue, influenza and yellow fever, differentiating between the agents of these illnesses remains a major challenge [18]. More often than not, non-specific treatment for malaria or bacterial infection are instituted, while Lassa fever is only suspected when a patient fails to improve with antimalarial and antibiotic therapy. Therefore, laboratory testing is needed to confirm the diagnosis [19]. Reliable laboratory diagnosis is crucial for the control and prevention of nosocomial and community-based transmission, as well as the initiation of ribavirin treatment. Various techniques are available for the diagnosis of Lassa fever, which includes viral culture, antigen and antibody detection assays and nucleic acid detection methods. Although, viral culture is the “gold standard” for diagnosis, its use is limited by the non-availability of a Biosafety level 4 (BSL-4) facility, the amount of monetary investment and time involved for such testing to be performed [18]. Antigen and antibody detection assays may provide a rapid diagnosis, but there is a high degree of false positive results once the antigenemia period has resolved in most patients [18]. Thus, nucleic acid-based assays such as the Reverse Transcriptase Polymerase Chain Reaction (RT-PCR) remains the available diagnostic method, which could be carried out using specimens that has been inactivated in a glove box and analyzed under Biosafety Level 2 (BSL-2) facilities [17,18]. In Nigeria, the availability of Lassa virus Reverse Transcriptase Polymerase Chain Reaction (RT-PCR) testing at the then Virology Unit Laboratory, College of Medicine of the University of Lagos (CMUL) since the early 2000s heralded the diagnosis and confirmation of Lassa Fever cases in various parts of the country [20,21]. However, only few laboratories have the capacity of diagnosing Lassa fever in the country, resulting in delays in diagnosis. The need for sample transportation from different parts of the country to only few laboratories which have the capacity to diagnose this infection is a major factor contributing to the delay in the diagnosis of Lassa fever. This diagnostic delay leads to delayed patient isolation, an increased potential for transmission to health care workers and family members, and delayed initiation of ribavirin therapy, thereby decreasing its beneficial effect [18]. Poor sample storage, transportation and handling may pose a safety hazard to the personnel involved in the sample movements, the laboratory staff and could also affect the integrity of the sample, thus decreasing the sensitivity of diagnostic assays. Ideally, a diagnostic assay would not only detect Lassa virus infection but would also screen for multiple other pathogens with similar clinical presentations endemic in West Africa such as yellow fever and dengue virus at the same time as practised in our laboratory in Lagos. Data on the laboratory diagnostic pattern using RT-PCR are limited in Nigeria, thus we aimed at providing information on the laboratory detection of Lassa virus in Nigeria.

## Methods

This was a retrospective cross-sectional analysis of 1,263 seasonal Lassa fever outbreaks samples from various states of the six geographic regions of Nigeria between years 2011 to 2017 submitted for Lassa Virus (LASV) RNA detection at the one of the National/ West African Health Organization (WAHO) Reference Laboratory for Viral Hemorrhagic Fevers and WHO Afro Reference Laboratory for Ebola Virus Disease in Lagos, Nigeria. All human blood samples were transported and received during the 7 years period using triple level packaging at the Virology Research Laboratory, Centre for Human and Zoonotic Virology (CHAZVY), College of Medicine of the University of Lagos/Lagos University Teaching Hospital. No ethics approval was obtained as the study was a diagnostic service evaluation. All data collated and presented in this manuscript were coded and treated with anonymity to protect the confidentiality of individuals with suspected cases of Lassa. All data and information were part of the routine surveillance during disease outbreaks for the identification of an infectious agent of public health concerns. All human blood samples were handled using personal protective equipment as recommended by the Centre for Disease Control and Prevention (CDC) [22] and were analyzed using Reverse Transcription-Polymerase Chain Reaction (RT-PCR) using specific primers for the segments of the s-gene of the Lassa virus, the 3’ non-coding region of the Dengue virus and the 5’ non-coding region of Yellow fever virus using an air tight glove box in a Biosafety Level-2 (BSL-2) facility. All data generated were evaluated and analyzed using the 21^st^ edition of SPSS statistical software.

## Results

**Figure 1:**
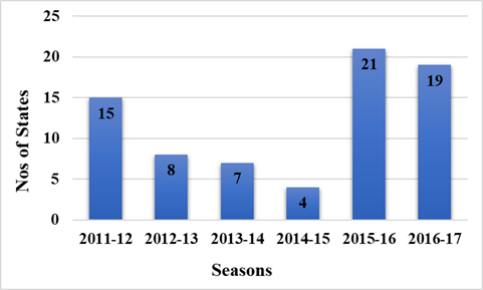
Number of states that sent samples to the virology unit, CRL by seasons

**Figure 2:**
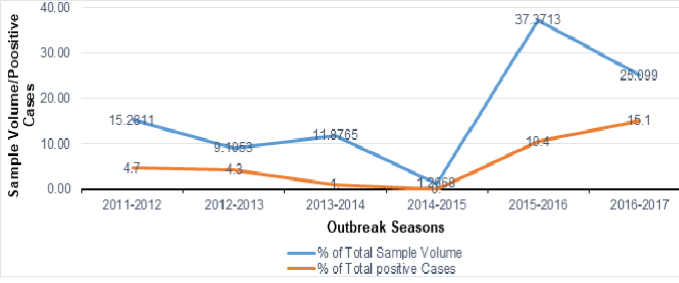
Percentage sample volume and percentage positive detected cases across outbreak seasons

**Figure 3:**
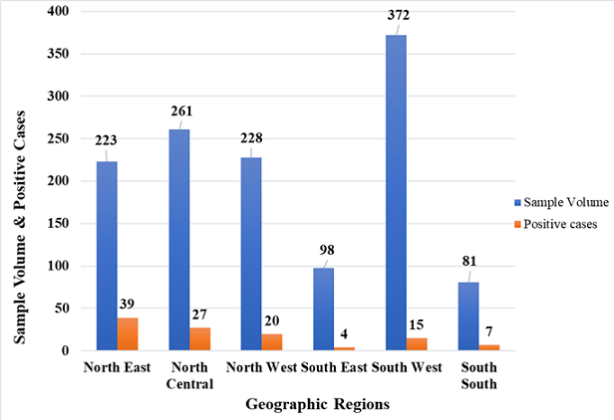
Sample volume and positive cases in the geographic regions of Nigeria

**Figure 4:**
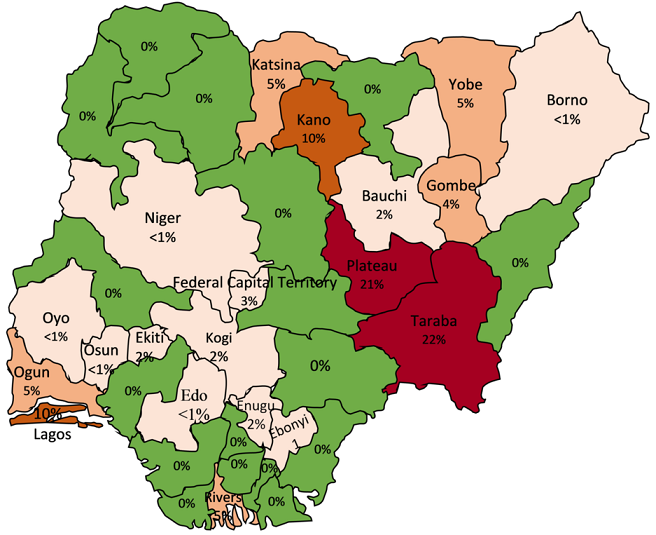
Distribution of Lassa fever confirmed cases in Nigeria

A total of 1,263 human blood samples comprising 43.4% males, 38.2% females and 18.4% without gender information were analyzed and evaluated in this study. The distribution of states that sent samples to facility revealed a gradual decrease in the number of states sending samples from an initial 15 states during the 2011-2012 outbreak to 4 states during the 2014-2015 outbreak (Figure 1). However, during the 2015-2016 and 2016-2017 Lassa outbreaks, there was an increase to 21 and 19 states respectively as shown in Figure 1. The RT-PCR test confirmed the presence of Lassa virus RNA in 112 (8.9%) individuals throughout the study period comprising 61 (54.4%) males, 48 (42.9%) females and 3 (2.7%) individuals without gender information without significance difference (p<0.05). All ages from less than 1 year to above 65 years were involved in the detected cases of Lassa. However, those aged between 18 and 49 years were the mostly affected that cuts across the years. There was a decline in the trend of lassa incidence from 4.7% in 2011/2012 to less than 1% by the 2014/2015, however, the country witnessed a resurgence of Lassa outbreaks from the 2015/2016 and 2016/2017 seasons with incidence rates of 10.4% and 15.1% respectively (Figure 2). Unlike before where RT-PCR confirmed Lassa positive cases were detected from few states, detection of Lassa was reported in more states during the 2015/2016 outbreak season in Nigeria. Confirmed Lassa RT-PCR positive cases were reported from 13 (61.9%) states (Kano, Abuja, Ekiti, Lagos, Ogun, Taraba, Rivers, Plateau, Gombe, Niger, Kogi, Katsina and Osun) out of the 21 states with suspected cases from the available data from our facility during the 2015/2016 outbreak season. While, Lassa activity was confirmed in 7 (36.8%) states (Kano, Ogun, Taraba, Rivers, Borno, Plateau and Gombe) of the 19 states with suspected cases during the 2016/2017 outbreak season sent to our facility. The Northern region of Nigeria has the highest number of suspected and confirmed cases of Lassa activities (Figure 3, Figure 4). However, the South Western region also witnessed an increased Lassa fever positivity rate of 13.4% of which Lagos and Ogun being the focal states of Lassa activity in the region (Figure 3, Figure 4).

## Discussion

Despite growing interest in Lassa fever, our knowledge of its actual incidence, burden, ecology, epidemiology and distribution of Lassa virus in West Africa is limited. The data in this study shows that Lassa fever cases are being detected in more states in Nigeria, which suggests that the infection is much more common than previously recognized as documented in this study [2]. Our study shows that the increase in LASV detection in one of the three LSV diagnostic centres in the country during this period indicates the changing geographic distribution of Lassa fever in Nigeria. The geographic location of the reservoirs and human cases within the country remains a major issue in determining the actual incidence and burden of LF as humans can move large distances after exposure before disease is detected. Other confounding factors that may affect the geographic distribution which also complicate diagnosis includes; the fairly elongated incubation period and non-specific clinical presentation very similar to other common and endemic African diseases; possible importation of LF from the states, countries and regions endemic for LF to other places not originally known for Lassa activity and the demonstrated evidence that there are other rodent reservoirs of LASV within the West African sub-region requires further evaluations [3,4,23-28]. The increasing detection patterns of LF as documented in this study was similar to the notable increase in suspected and confirmed LF outbreaks across a growing area in the West African sub-region. Regional environmental changes particularly in Nigeria may have impacted LF ecology and epidemiology in several ways. Nigeria and other West African countries have experienced profound environmental, socioeconomic and demographic shifts in recent decades, including population growth, land use change (including deforestation, mining and commercial agricultural expansion), bush-meat extraction, urbanization and growing transport connectivity [28,29]. The region has also been forecasted to experience strong climate change effects, impacting crop yields alongside other important ecosystem services [30]. Such anthropogenic processes are thought to be major drivers of zoonotic and vector-borne diseases [31]. Thus, proper sensitization of the community on the modes of transmission and preventive measures should be implemented to reduce burden of disease. Adequate training and retraining of healthcare workers are urgently required. Establishing well-equipped clinic, laboratories and research centres would help in the prompt containment, diagnosis and treatment of Lassa fever. These established the need for heightening the continued surveillance for Lassa in our environment.

## Conclusion

The findings from this study attest to the changing geographic distributions and spread of Lassa fever in the country. The actual picture of the disease's true incidence and distribution in Nigeria and the West African region, with consequences for surveillance, diagnosis, treatment and disease management still needs to be elucidated. Laboratory capacity for the diagnosis and identification of geographical variation and genetic correlates of LASV strain in all the geo-political regions of Nigeria is hereby emphasized.

### What is known about this topic

- The diagnosis of Lassa fever which presents with signs and symptoms related to other febrile endemic diseases remains a major challenge in a resource limited setting like Nigeria;
- The nucleic acid-based assay; Reverse Transcription-Polymerase Chain Reaction (RT-PCR) remains the available diagnostic method;
- Prompt analysis and return of test results within specified test turn-around time (TAT) had greatly influenced the outcomes of Lassa fever cases in Nigeria.

### What this study adds

- Data presented here confirmed the resurgence of Lassa fever epidemic since the 2015-2016 outbreaks till date;
- This study highlights the changing geographic distributions of Lassa fever outbreaks in Nigeria with increasing numbers of states involved;
- The study also brings to light the need for the establishment of other testing facilities within the endemic regions for prompt diagnosis of Lassa fever in the country.

## Competing interests

The authors declare no competing interests.
